# Novel Protocol for the Chemical Synthesis of Crustacean Hyperglycemic Hormone Analogues — An Efficient Experimental Tool for Studying Their Functions

**DOI:** 10.1371/journal.pone.0030052

**Published:** 2012-01-11

**Authors:** Alessandro Mosco, Vientsislav Zlatev, Corrado Guarnaccia, Sándor Pongor, Antonella Campanella, Sotir Zahariev, Piero G. Giulianini

**Affiliations:** 1 Department of Life Sciences, University of Trieste, Trieste, Italy; 2 International Centre for Genetic Engineering and Biotechnology, AREA Science Park, Trieste, Italy; International Centre for Genetic Engineering and Biotechnology, Italy

## Abstract

The crustacean Hyperglycemic Hormone (cHH) is present in many decapods in different isoforms, whose specific biological functions are still poorly understood. Here we report on the first chemical synthesis of three distinct isoforms of the cHH of *Astacus leptodactylus* carried out by solid phase peptide synthesis coupled to native chemical ligation. The synthetic 72 amino acid long peptide amides, containing L- or D-Phe^3^ and (Glp^1^, D-Phe^3^) were tested for their biological activity by means of homologous *in vivo* bioassays. The hyperglycemic activity of the D-isoforms was significantly higher than that of the L-isoform, while the presence of the N-terminal Glp residue had no influence on the peptide activity. The results show that the presence of D-Phe^3^ modifies the cHH functionality, contributing to the diversification of the hormone pool.

## Introduction

The crustacean Hyperglycemic Hormone is a neuropeptide synthesized and secreted by the X organ-sinus gland complex (XO-SG) located in the eyestalks of decapod crustaceans. Its main role concerns the regulation of the hemolymphatic glucose level, but additional functions include growth control, reproduction, ion balance, stress responses [1]. cHH belongs to a family of crustacean peptides that includes the Molt Inhibiting Hormone (MIH), the Vitellogenin Inhibiting Hormone (VIH) and the Mandibular Organ Inhibiting Hormone (MOIH). All these peptides are structurally related, having six conserved cysteines that form three intramolecular disulfide bridges [2]. The family can be further classified into two subfamilies on the basis of the primary structure and the preprohormone peptide organization. The cHH subfamily or type I possesses a cryptic sequence of unknown function, named cHH precursor-related peptide, in the unprocessed precursor [3], while the MIH/VIH/MOIH subfamily or type II peptides that are longer and more variable in length, have a Gly at position 12 and lack the cHH precursor-related peptide [4] [5] [6]. Mature cHHs have a length of 72 to 73 amino acid residues, and an homology among the different species ranging from 40 to 99% [7]. They have an amidated C-terminus and, in many cases, pyroglutamate as N-terminal blocking group [1] [2]. Moreover, the phenylalanyl residue at position 3 of the sequence can be found in either L- or D-configuration [8].

C-terminal amidation is the most significant post-translational processing, because of its influence on cHH bioactivity, and the lower functionality showed by non-amidated peptides can be explained by a lower binding affinity to the cHH receptor due to their negative C-terminus charge [9] [10].

Another post-translational modification concerns the cyclization of the Glu residue at the N-terminal end, which is found in cHHs of brachyuran crab species and crayfish, but not in shrimps [11]. The amino acid sequence of both N-terminus blocked and free cHHs is almost identical, whereas the N-terminal residue can be glutamine or pyroglutamate. In *C. maenas* both isoforms proved to possess a similar biological activity and the same hemolymphatic clearance rates. Thus seems that N-terminal cyclization has no obvious biological function [12] [13], but it may well be that the presence of a blocked N-terminus protects the cHH against peptidases, extending its half-life like in other peptides [14].

D-amino acids have been found only in the peptides of some invertebrates and amphibians. These include a toxin from a spider venom, a snail neuropetide, some crustacean hormones of the cHH family, and opiate and antimicrobial peptides from amphibian skin [15]. To date L and D stereoisomers have been reported only for the cHH of Astacoidea (*Astacus leptodactylus*, *Cherax destructor*, *H. americanus*, *Orconectes limosus*, *Procambarus bouvieri, Procambarus clarkii*), the stereoinversion concerning always the Phe^3^ [16] [8] [17]–[20]. Little is known about the role of isomerization and if the two stereoisomers have the same functions or exhibit distinctive bioactivities. The presence of D-Phe^3^ seems to confer a higher hyperglycemic activity to the cHH. In an *in vivo* heterologous bioassay, *O. limosus* was injected with L- or D-cHHA extracted from SG of *H. americanus*, and purified by reverse-phase high performance liquid chromatography (RP-HPLC). The peak value of glycemia was in the same range for both isoforms, 30.5±6.7 mg/dL and 34.3±5.6 for the L and D form respectively, but the time course was different, with the D-cHHA inducing a later response, having the maximal hyperglycemic peak at 3–4 h instead of 2 h as for the L-cHHA, and a slower return to the base-line, being the hyperglycemic effect still detectable at 8 h [8]. A similar time course was found in *A. leptodactylus* after injection of homologous D-cHH, purified by RP-HPLC from a sinus gland extract, which induced a higher hyperglycemia and had an extended response, the hemolymph glucose level being significantly higher both at 3 h and 8 h, when compared to the concentration elicited by the L-cHH. The same study proved that cHH is involved in the control of osmoregulation, and that the different stereoisomers may have different target tissues and/or receptors. Indeed, all the purified cHHs were able to increase hemolymph Na^+^ concentration, but only D-cHH induced a significant raise in hemolymph osmolality [19]. The higher efficacy of the D isomer in exerting a biological activity was proved also for the native cHH of *P. clarkii*, purified from the XO-SG complex. In *in vitro* experiments it was shown that the cHH is able to inhibit ecdysteroid biosynthesis in cultured Y-organs, suggesting that this neuropetide may play a role in the crustacean molting process. Of the two isoforms tested, the D-cHH showed a ten fold higher inhibitory effect on ecdysteroidogenesis as well as being more potent in inducing hyperglygemia [16]. On the contrary, in the closely related Mexican crayfish *P. bouvieri*, whose cHH share an identical amino acid sequence with those of *P. clarkii*, the two isoforms showed the same ability to induce hyperglycemia in destalked animals [17].

The present study describes the hyperglycemic activity of 3 analogues of cHH of the crayfish *A. leptodactylus*, prepared for the first time by chemical synthesis, demonstrating the attractive possibility to synthetically produce milligram amounts of cHH in a specific desired form for future studies deciphering its functions.

## Results

### Peptide synthesis

**Figure 1:**
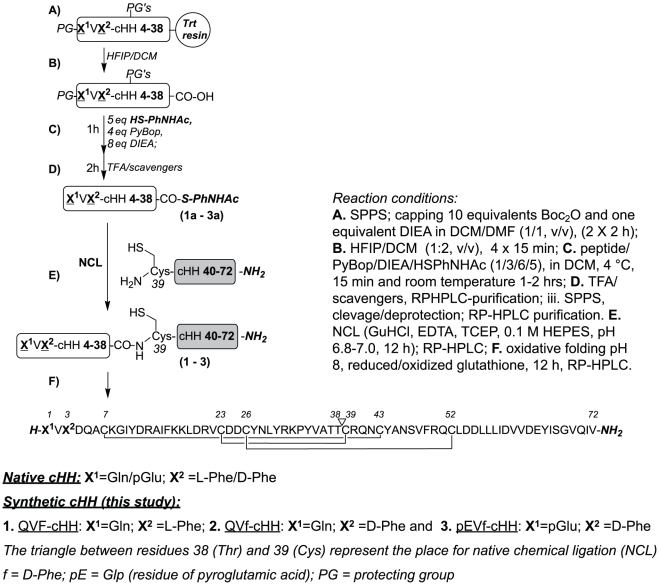
Scheme of the SPPS coupled to NCL to obtain the full length peptides. The leading segments containing a C-terminal thioester, QVF-cHH4-38, QVdF-cHH4-38, pEVdF-cHH4-38, and the cHH39-72 following segment with a free N-terminal cysteine were synthesized by SPPS. The NCL reaction between the leading and following segments returned the full length 72mer cHH isomers, which were further subjected to oxidative folding to obtain the folded peptides.

The sequence of cHH1-72 and of its isomers (Fig. 1) contain several potentially problematic sites for the SPPS with Fmoc/tBu strategy. It contains six cysteine residues (prone to racemization), nine aspartic acid residues (possible aspartimide formation, followed by isomerisation, racemization and piperidide formation) and a very hydrophobic region in the C-terminus part since 10 residues on the last 17 aa (56–72) are Leu, Ile, or Val (possible poor couplings and Fmoc-deprotection, due to secondary structure formation on the resin).

**Figure 2:**
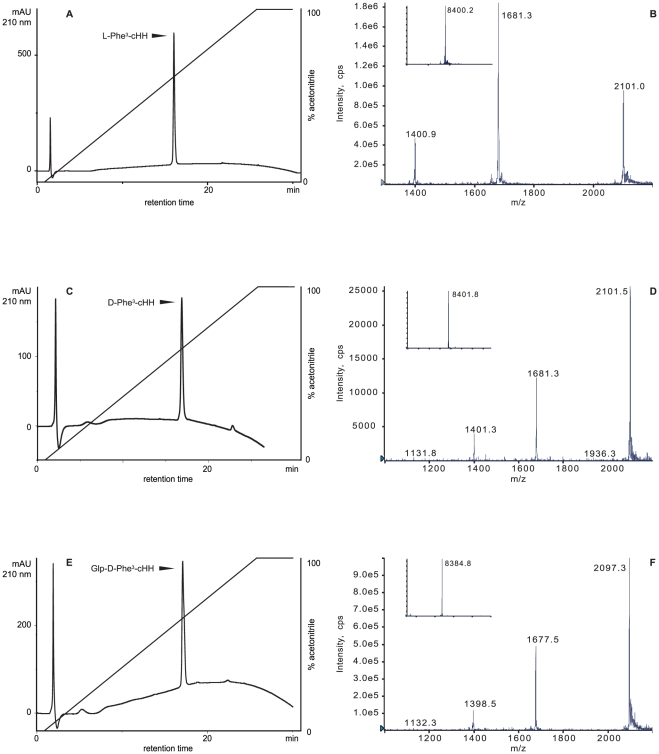
RP-HPLC profiles and ESI-MS spectra of the synthesized cHH isomers. Reverse Phase HPLC profiles of the purified refolded synthetic polypeptides and their corresponding ESI-MS multiply charged ion and deconvoluted spectra (insets): L-Phe^3^-cHH (A and B), D-Phe^3^-cHH (C and D), Glp-D-Phe^3^-cHH (E and F). Mobile Phase A: 0.1% TFA in water. Mobile Phase B: 0.1% TFA in MeCN. Gradient: 0–100% B over 25 min. Column: Gemini 5 C18 2.0×150 mm.

In our approach the sequence was divided in two parts, cHH1-38 and cHH39-72 in order to use a native chemical ligation reaction at Cys^39^ to obtain the full length 72mer peptide (Fig. 1) First, side chain and N-terminus Boc-protected cHH1-38 peptides (Fig. 1, A) were synthesized by SPPS using Fmoc-methodology on a very acid labile chlorotrityl resin. Second, protected peptide carboxylic acids were cleaved from the resin with hexafluoroisopropanol (HFIP) in dichloromethane (DCM) (Fig. 1, B). At the end (Fig. 1, C), the crude protected peptides were esterified to corresponding 4-acetamidophenyl-thioesters, according to published procedure [21]. After acid deprotection (Fig. 1, D), with thiol free cleavage mixture, all 3 peptides thioaryl esters were purified by RP-HPLC. C-terminal cHH39-72 peptide amide was synthesized on Tentagel Sieber amide resin (Merck), a resin for Fmoc SPPS derivatized by xanhydrylamine linker which gives peptide amide fragments upon cleavage with trifluoroacetic acid (TFA) [22]. After cleavage and deprotectection [TFA, triisopropylsilane (TIPS), H_2_O, 3,6-dioxa-1,8-octanedithiol (DODT), phenol, (81%/3%/3%/8%/5%) for 4 h] the C-terminal peptide amide was purified by semipreparative RP-HPLC. The purity/identity of all peptides was verified by liquid chromatography electrospay ionization mass spectrometry (ESI-MS) (Table 1, Fig. 2). Even though the synthesis of cHH39-72 went smoothly (the purity of crude peptide was >55%), the deprotected and purified peptide possesses a very low solubility in aqueous buffers (due to its high hydrophobic AAs content), which complicates both purification/recovery and followed step of NCL reactions (Fig. 1, E). Despite the several solubilization conditions tried [guanidine hydrochloride (GuHCl), urea, sodium dodecyl sulfate, 2,2,2-trifluoroethanol, HFIP, and its mixtures] a substantial cHH39-72 precipitation occurred in all NCL conditions tested, negatively affecting the final yields of purified and folded (see experimental part) cHH1-72 isomers. Different approaches such as introducing on C-terminus solubilizing cleavable tails are actually under evaluation. Purified linear 72 amino acids long peptides were folded (Fig. 1, F) in basic condition in the presence of oxidized/reduced glutathione or Cys/Cys2 and purified to homogeneity by RP-HPLC with yields between 1 and 3%.

**Table 1:** ESI-MS data of cHH analogues (**1**–**3**), thioesters (**1a**–**3a**) and cHH (39–72)-amide.

	cHH (1–72)		cHH aryl thioester (1–38)	cHH (39–72)-amide
	calc.(av.)/found (Da)		calc.(av.)/found (Da)	calc.(av.)/found (Da)
1	L-Phe^3^-cHH:	1a	QVF-cHH-4-38:	
	8407.7/8406.0		4670.4/4669.2	
2	D-Phe^3^-cHH:	2a	QVf-cHH-4-38:	3904.5/3903.0
	8407.7/8406.2		4670.4/4669.9	
3	Glp-D-Phe^3^-cHH:	3a	pEVf-cHH-4-38:	
	8390.7/8385.0		4653.4/4651.2	

### Bioassays

**Figure 3:**
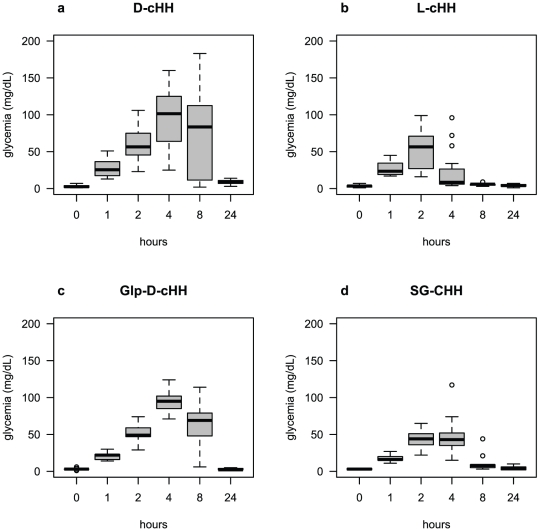
Time course of the induced glycemia after injection of the synthetic and wild type cHHs. a) Time course of hemolymph glycemia after injection of 1.7 pmol/g live weight of D-cHH. The maximum peak of 95.4±8.1 mg/dL glucose is reached after 4 h, high values lasting at 8 h (76±12.1 mg/dL), when the maximum values were also recorded, and the glycemia returning to its nearby basal level, 8.9±1 mg/dL, after 24 h. N = 20. b) Time course of hemolymph glycemia after injection of 1.7 pmol/g live weight of L-cHH. L-cHH induced a slower hyperglycemic response with the maximum peak of 52±6.5 mg/dL glucose after 2 h, and the glycemia returning to its nearby basal level, 5.5±0.4 mg/dL, after 8 h. N = 16. c) Time course of hemolymph glycemia after injection of 1.7 pmol/g live weight of Glp-D-cHH. The time course of Glp-D-cHH shows the stronger hyperglycemic response of 96.1±5.6 mg/dL glucose after 4 h, high values lasting at 8 h (62.9±11.8 mg/dL) and the glycemia returning to its basal level, 2.9±0.5 mg/dL, after 24 h. N = 9. d) Time course of hemolymph glycemia after injection of 1.7 pmol/g live weight of SG-cHH. The hyperglycemic time course after the injection of 1.7 pmol/g live weight of purified SG-cHH was comparable to that of synthetic D-cHH and Glp-D-cHH peptides, but the maximum (44±4.5 mg/dL) was reached after 2 h and the glycemia returning to its basal level, 5±1.1 mg/dL after 24 h. N = 9.

The biological assays on *A. leptodactylus* were performed as time-course experiments to assess potential biological activity differences among the three chiral isoforms of the synthetic peptide (Fig. 1, **1**, **2** and **3**) and to compare them to the native hormone. The two chiral synthetic isoforms of cHH showed significant different hyperglycemic responses in term of both time course/kinetic pattern and absolute values of circulating glucose (Fig. 3).

**Figure 4:**
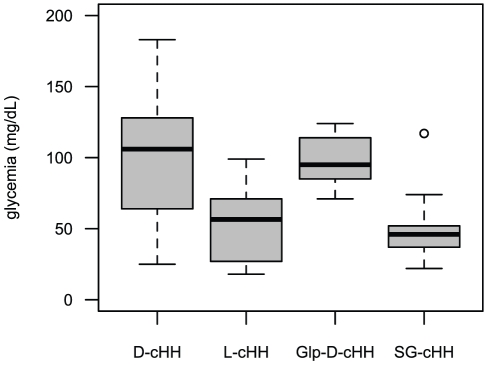
Boxplots of the maximum glucose values recorded for the synthetic and native peptides. Both D-cHH and Glp-D-cHH induced a strong hyperglycemia with a mean peak glucose concentration of, respectively, 100.5±9.3 and 98.7±6 mg/dL, while the hyperglycemic effect of GS-cHH and of L-cHH was lower, with a mean of max glycemia of 52.7±9.4 mg/dL and of 52.4±6.4 mg/dL respectively.

Injection of 1.7 pmol/g live weight of each of the three analogue peptides revealed the following results: synthetic D-cHH induced a quick hyperglycemic response already detectable after 1 h and a strong hyperglycemic response with the maximum peak of 95.4±8.1 mg/dL glucose after 4 h, high values lasting at 8 h (76±12.1 mg/dL) and glycemia returning to its nearby basal level, 8.9±1 mg/dL, after 24 h (Fig. 3, a). Glp-D-cHH mirrored the time course of D-cHH, with the strongest hyperglycemic response of 96.1±5.6 mg/dL glucose after 4 h, high values lasting at 8 h (62.9±11.8 mg/dL) and the glycemia returning to its basal level, 2.9±0.5 mg/dL, after 24 h (Fig. 3, c). L-cHH induced a lower hyperglycemic response with the maximum peak of 52±6.5 mg/dL glucose after 2 h, and the glycemia returning to its nearby basal level, 5.5±0.4 mg/dL, after 8 h (Fig. 3, b). Injection of the purified SG-cHH was comparable to that of the synthetic L-cHH, the maximum (44±4.5 mg/dL) being reached after 2 h, and the glycemia returning to its basal level, 5±1.1 mg/dL after 24 h (Fig. 3, d). The comparison of the glycemia elicited by each of the analogues during the first 8 hours post-injection revealed significant differences between time points (Kruskal-Wallis *p*<0.01) and the Bonferroni adjusted *p* values of post-hoc pairwise comparisons at different times compared to initial values were significant. At 24 h, only the hyperglycemia induced by the D-cHH was still significantly different from that of time 0 h. The comparison among the glycemic values elicited by the different peptides 4 or 8 hours post-injection revealed significant differences (4 h – Kruskal-Wallis chi-squared = 29.5491, df = 3, *p* = 1.72⋅10^−6^ ***; 8 h – Kruskal-Wallis chi-squared = 21.7912, df = 3, *p* = 7.209⋅10^−5^ ***). The Bonferroni adjusted *p* values of post hoc pairwise comparisons revealed two peptide groups, D-cHH and Glp-D-cHH on one hand eliciting significantly higher glycemic response than L-cHH and SG-cHH on the other hand. To evaluate the overall hyperglycemic potentiality of the three synthetic peptides and the native one, the maximum values recorded in the experiments for each specimen from 1 h to 8 h were plotted in Fig. 4. The D-cHH and the Glp-D-cHH (means of max glycemia of, respectively, 100.5±9.3 and 98.7±6 mg/dL) showed again their higher capacity to mobilize the glucose compared to both L-cHH and SG-cHH (means of max glycemia of, respectively, 52.4±4 and 52.7±9.4 mg/dL).

## Discussion

The cHH plays a major role in controlling the hemolymphatic glucose level, but it also has significance in regulating molt, reproduction and homeostasis, nevertheless the putative distinct functions exerted by the different circulating cHH forms are still poorly understood. To study the biological role of the cHH family peptides, large amounts of them are required, and in the past years different approaches were tried in order to obtain sufficient peptide quantities. To achieve this purpose different technologies were used: extraction from natural sources [23] [24] [25], production by recombinant DNA technology [26] [27] [10], and by chemical synthesis [28].

The purification of native neuropeptides by RP-HPLC has a low yield, about 2–4 µg (270–489 pmol) per sinus gland in *C. maenas* [29], but the invaluable advantage is to obtain naturally folded peptides, complete of all the post-translational modifications. DNA recombinant technology proved to be useful to obtain crustacean peptides in greater quantities to enable further investigation of their physiological functions. Recombinant cHHs from *Metapenaeus ensis* [30], *Nephrops norvegicus* [31], *Penaeus japonicus* [9], *Macrobrachium rosenbergii* [32], and *A. leptodactylus* [10], were produced using bacterial expression systems. All these works reported the insertion of fusion tags for purification of target proteins by affinity chromatography. These additional sequences may interfere with the receptor binding which results in a lowered biological activity [9]. Moreover, recombinant peptides are never obtained with an amidated C-terminus, thus having a lower functionality. Therefore, this important post-translational modification must be added later through a specific enzymatic reaction [9] [10], while another post-translational modification, the pyroglutamilation of the N-terminus, is prevented by the presence of N-terminal tags. To overcome the problems posed by peptide expression in *Escherichia coli*, an eukaryotic expression system was developed. The cHH from *Penaeus monodon* was successfully expressed in the yeast *Pichia pastoris*, from which it was secreted to the culture medium already correctly refolded, and from it directly purified by RP-HPLC. The recombinant Pem-cHH was capable of inducing hyperglycemia in eyestalk-ablated prawns, although with a lower potency compared with the native peptide, due probably to the lack of C-terminal amidation [33].

Chemical synthesis enables a level of control on protein composition that greatly exceeds that attainable with ribosome-mediated biosynthesis, allowing post-translational modifications like C- and N-terminus blockage and insertion of D-amino acids, while native chemical ligation allows to overcome the limit of the peptide length posed by SPPS [34]. This approach was applied to synthesize the MIH from *P. clarkii.* The synthetic peptide was comparable to the sinus gland extracted Prc-MIH in the inhibition of ecdysteroid secretion by *in vitro* cultured Y-organs [28].

Our study was aimed at the production of several isoforms of the cHH of *A. leptodactylus*, by means of SPPS coupled with the native chemical ligation, in order to verify the effect that N-terminus pyroglutamylation and isomerization of the Phe^3^ have on peptide functionality through *in vivo* homologous bioassays.

Changes in amino acid chirality add more variety to the pool of synthesized peptides. The presence of a D-amino acid may lead to structural modifications as the peculiar type II′β-turn structure found in the snail Gly-D-Phe-Ala sequence of achatin I [35] or the β-turn of amphibian opioid peptides that is involved in the receptor recognition [36]. The L-isomers of these peptides do not bind to the corresponding receptors, thus having no biological function. In other cases D-peptides were found to be far more potent in eliciting a biological response. Frog dermorphin, when injected into the rat brain, is about one thousand more potent then morphine in inducing long-lasting analgesia [37]. This behaviour may be also the result of a resistance to peptidases, as L-dermorphin is rapidly degraded, while the D-peptide is not hydrolyzed [15].

Our study shows that D-cHH was more potent than L-cHH in inducing glycemia, with a quick response detectable after 1 h and the maximum peak of 95.4±8.1 mg/dL glucose after 4 h from injection. On the contrary, L-cHH had its maximum peak at 2 h after injection, with hyperglycemic peak of 52±6.5 mg/dL. The superiority of D-cHH over the L-counterpart is probably due to a change in the secondary structure of the peptide that may increase the cHH affinity for receptors located on its target organs. This behavior is in agreement with previous studies. Indeed, D-cHH purified from SG of *A. leptodactylus* exhibited a higher hyperglycemic response compared to the L-isomer [19]. The hyperglycemic effect triggered by D-cHH was extended in time: the glycemia recorded at 24 h was still significantly elevated (*p* = 1.1⋅10^−3^) respect to basal levels (time 0). This result is surprising, if we consider that cHH is cleared from the hemolymph quite dynamically, being of 10 min the half life reported in *C. maenas* [38]. The prolonged effect could be due to a slower dissociation constant from the receptor, as consequence of the conformational variation in 3D structure of D-isomers, or due to a higher resistance to peptidases. A low, but significant hyperglycemia at 24 h after injection with native D-cHH was already found in *A. leptodactylus* [19], an effect corroborated by our findings. The different hyperglycemic time-course of the L and D isomers was also shown in a heterologous bioassay, where the injection of D-cHH from *H. americanus* into *O. limosus* produced a longer hyperglycemic response than that caused by equal doses of L-cHH, even if the maximal hyperglycemic responses were comparable [8]. The Glp-D-cHH was more potent than the N-terminus blocked native hormone of *A. leptodactylus*, inducing a higher hyperglycemic outcome. Native cHH circulates in the hemolymph as a mixture of L- and D-isoforms, of which the D-cHH is the less abundant, in *C. destructor* amounting to 30–40% of the L-isoform [20]. The hemolymph content of cHH reflects the ratio of cells synthesizing the two isomers. In the X-organ of the crayfish *O. limosus*, a cluster of approximately 30 cells producing cHH was identified, of which only about 8 cells synthesize the D-cHH [18]. Therefore, it is conceivable that the minor activity of the native peptide extract was due to the higher proportion of the L-isoform which, as we have seen, has a lower activity. Our results show that the isomerization of the Phe^3^ affects the cHH functionality modifying its capability to mobilize the glucose reserves, and being cHH a pleiotropic hormone involved, besides glucose regulation, in secretion of digestive enzymes [39], lipid metabolism [40], osmoregulation [19] [41], growth [29] [42], and reproduction [43] [44], it can be also assumed that the two isoforms may play different roles in various physiological contexts. In the crayfish *P. clarkii* both stereoisomers exhibit hyperglycemic activity, but the D-isomer showed a more potent inhibition of ecdysteroid synthesis in *in vitro* cultured Y-organ [16]. The involvement of cHH in the control of osmoregulation was proved in the crab *P. marmoratus* where cHH extracted from SG increased the Na^+^ influx in perfused posterior gills from crabs acclimated to diluted seawater [45]. The ability of this neuropeptide to exert an effect on osmolality and Na^+^ concentration of the hemolymph was proved in *in vivo* bioassays on *A. leptodactylus*. The injection of D-cHH raised both hemolymph osmolality and Na^+^ concentration after 24 h, while L-cHH had no significant effect on osmolality, but increased Na^+^ concentration, however with a lower rise [19].

Besides isomerization, we studied the effect of another post-translational modification, pyroglutamylation from the N-terminal glutamine residue of cHH. It has been already proved in crabs that pyroglutamyl-blocked and unblocked cHHs exhibited identical ability to induce hyperglycemia *in vivo* and to inhibit ecdysteroidogenesis *in vitro*, and showed similar clearance/degradation rates in the hemolymph. Both cHH variants are present in the sinus glands at a constant ratio, the blocked cHH being the major form, and the same proportion is maintained in the hemolymph [12]. In a study of the biosynthesis of cHH performed as time-course experiments with pulse-chase incubations, on *in vitro* cultured X-organ SG complexes from *O. limosus*, cyclization resulted incomplete as glutaminyl L-cHH represented about 30% of the blocked peptide. Because cyclization of 70–80% of the cHH occurs within 8 h, then remaining constant, it was supposed that this modification is catalyzed by a putative glutaminyl cyclase during the axonal transport [46]. Our results confirm the bioactivity data already found in crabs, because the hyperglycemic activity showed by blocked cHH, Glp-D-cHH, was not significantly different (*p*>0.9) at any time of the time course from that of D-cHH, whose N-terminus is free. The similar behavior of these two forms suggests that the pyroglutamate block at the N-terminus has no biological function, at least with respect to hyperglycemic activity or to peptide half-life, though it is widely accepted that N-terminal blocked peptides are more resistant to aminopeptidases [14].

Among the protein post-translational modifications, which enrich the diversity of the peptides encoded by the same gene, the fascinating modification of chirality is the less understood. Our data indicate that SPPS coupled to native chemical ligation is a suitable strategy for the synthesis of crustacean peptides of the cHH family, being the only one that allows the introduction of all the post-translational modifications necessary to obtain a peptide identical to the native hormone, and therefore it is an appropriate alternative to DNA recombinant technology. The optimization of the present synthesis protocol can make available cHH peptides for large scale *in vivo* and *in vitro* experiments that could lead to a better understanding of cHH role and structure-activity relationships.

## Materials and Methods

### Reagents

9-Fluorenylmethoxycarbonyl (Fmoc) protected amino acid building blocks were ordered from Novabiochem, Bachem, and Fluka. Resins for SPPS were from Novabiochem and Fluka. Benzotriazol-1yl-oxy-tris-(pyrrolidino)-phosphonium-hexafluorophosphate (PyBOP) and 2-(6-Chloro-1H-benzotriazole-1-yl)-1,1,3,3-tetramethylaminium hexafluorophosphate (HCTU) activators were from NovaBiochem. Anhydrous N,N′-dimethylformamide (DMF) and DCM were from Romil; phenol was from AnalR; piperidine (PIP) and HFIP were from IRIS Biotech; N-diisopropylethylamine (DIPEA) and TFA were from Biosolve; TIPS from Alfa Aesar; DODT, diethylether, petroleum ether and dichloroethane (DCE) were from Sigma; 4-acetamidothiophenol (HSPhNHAc) was from Merck.

### Peptide synthesis

Peptides were synthesized at 0.1 mmol scale by solid phase method using Fmoc/tBu chemistry. Both N- and side chains protected peptide carboxylic acids from N-terminal sequence of cHHs (1–38) were assembled on Fmoc-Thr(But)-TGT-resin (Fluka, substitution ∼0.2 mmoles/g). cHH39-72 amide was synthesized at 0.3 mmol scale on TentaGel Sieber amide resin (Novabiochem, 0.2 mmol/g).

Side chain protecting groups were: Trityl (Trt for Asn, Cys, Gln), t-Butyl (tBu for Ser, Thr), t-Butyloxycarbonyl (Boc for Lys, Tyr, Trp), tert-Butylester (OtBu for Asp, Glu), 2,2,4,6,7-pentamethyldihydro-benzofuran-5-sulfonyl (Pbf for Arg). The Fmoc amino acid (four molar excess with respect to the initial resin substitution), the coupling reagent (HCTU), and the base (DIPEA), in a ratio of 1∶1∶2, were dissolved in DMF to give a final amino acid concentration of 0.3 M. Systematic double coupling was adopted to add residues using PyBOP as coupling reagent instead of HCTU. Fmoc protecting groups were removed by a 20% (v/v) solution of PIP in DMF containing 0.1 M 1-hydroxybenzotriazole to decrease aspartimide formation. In order to minimize Cys racemization, 4 equivalents for 2 h of Fmoc-Cys(Trt)-OPfp in DMF was employed in the first coupling. The second coupling was performed for 1 h with four molar excess of Fmoc-Cys(Trt)-OH, HCTU, 2,6-dimethylpyridine, in 1∶1∶2 ratio as a 0.3 M solution in DCE/DMF 1∶1 (v/v), as described [47].

Cleavage and side chain deprotection of cHH39-72 peptide amide resin was performed for 4 h with 20 mL/g resin of TIPS/H_2_O/DODT/phenol/TFA (3%/3%/8%/5%/81%), followed by precipitation and washing with diethyl ether and drying. Crude cHH39-72 peptide amide were reduced by 10 molar excess tris(2-carboxyethyl)phosphine (TCEP) in 0.1 M Tris pH 8 with 6 M GuHCl and purified by semipraparative RP-HPLC (Jupiter C4 10×250 mm, Phenomenex) using 0.5% gradient slope from 0.1% TFA in water (mobile phase A) to 0.1% TFA in 98% acetonitrile (MeCN) (mobile phase B).

Cleavage of all three protected cHH1-38 peptide isoforms was performed separately by 33% HFIP in DCM (4 times with 2 resin volume for 15 min). The solvents were evaporated, the rest was washed with Et2O/hexane (1/3) and dried. Thioester formation of cHH1-38 isoforms was performed according published procedure [21]. Briefly, 200 mg (∼0.027 mmol) each of protected cHH1-38 was suspended in 1 ml DCM containing 27.9 mg (0.216 mmol, 8 eq.) DIPEA, mixed for 2 min at room temperature under nitrogen, then cooled to 4°C; dry PyBop 56.2 mg (0.108 mmol, 4 eq.) was added, mixed vigorously for 0.5 min and then 22.7 mg (0.135 mmol, 5 eq.) dry HSPhNHAc (purity >99%, freshly prepared by TCEP reduction) was added under nitrogen. The reaction mixture was vigorously mixed for additional 4 min and then occasionally in total for 2 h. The solvent was evaporated and the rest (oil) was mixed with 3 ml Et2O and 6 ml petroleum ether, the solid was centrifuged, washed again with 2 ml petroleum ether and dried *in vacuo*. The crude peptide thioesters were fully deprotected for 3 h with H_2_O/TIPS/Phenol/TFA (2.5%/2.5%/5%/90%, 20 mL/g resin) and then precipitated/washed with diethyl ether. The crude peptide thioesters were dissolved and purified by semipreparative RP HPLC as described above. Pure (according ESI-MS, API 150EX, ABSciex) fractions were pooled and feeze-dried.

### Native chemical ligation

The native chemical ligations of the thioester peptides cHH1-38 with cHH39-72 were performed dissolving the two peptide components at concentration ∼5–10 mg/mL in ligation buffer [(0.5 M 4-(2-hydroxyethyl)-1-piperazineethanesulfonic acid (HEPES), 6 M GuHCl, 50 mM TCEP, 20 mM ethylenediaminetetraacetic acid (EDTA)] at pH 6.8–7.0. The N-terminal peptide was used in slight molar excess (∼10%) with respect to the counterpart. The reactions went overnight at RT under gentle stirring.

### Purification and oxidative folding

The NCL reaction mixtures were analyzed by LC-MS and purified by RP-HPLC in two different mobile phase systems. Linear reduced L-cHH and D-cHH were purified from the NCL mix by RP-HPLC using the classical acidic (0.1% TFA) mobile phase system on a semipreparative column Jupiter C4 10×250 mm (Phenomenex) using a gradient of 0.5% B/min and the product was eluted at ∼40% MeCN. The pure fractions (according ESI-MS) of L-cHH and D-cHH were pooled and freeze dried. The peptides were re-dissolved at 0.1 mg/ml and subjected to oxidative folding in 0.25 M Tris-HCl, 5 mM reduced glutathione, 1 mM oxidized glutathione, 1 mM EDTA buffer adjusted at pH 8 under argon. The refolded L-cHH and D-cHH were purified by RP-HPLC on a semipreparative column Jupiter C4 10×250 mm using a gradient of 0.5% B/min. The product was eluted at ∼38% MeCN. The Glp-D-cHH NCL reaction mixture was instead purified using the same column but in neutral pH conditions (Mobile Phase A was 20 mM triethylammonium acetate in water, Mobile Phase B was 20 mM triethylammonium acetate, 80% acetonitrile) and a gradient of 0.75% B/min from 15% to 60% B. Pure (according to ESI-MS) fractions were pooled and freeze-dried. Purified (according to ESI-MS) fraction of Glp-D-cHH at pH 7 was diluted to 0.1 mg/mL, directly folded by air oxidation overnight under gentle stirring and then freeze dried. The loss of 6 Da in the molecular masses indicating the completeness of the three disulfide bonds formation was verified by ESI-MS in all cases.

### Extraction and purification of native Asl-cHH

Sinus glands from 40 eyestalks of *A. leptodactylus* were collected and 100 µL of extraction solution (90% MetOH, 9% acetic acid, 1% H_2_O) was added. After sonication, the sample was centrifuged at 12 000× g for 10 min at 4°C, and the supernatant collected. The pellet was resuspended in 100 µL of the extraction solution, centrifuged again, and the two supernatants were mixed together. The extract was purified on a HPLC system (Gilson) equipped with a Zorbax SB-C18 4.6×150 mm column from Agilent Technologies Inc. (DE, USA) thermostated at 25°C. Mobile Phase A was 0.1% TFA in water, Mobile Phase B was 0.1% TFA in acetonitrile. The separation was done using a gradient of 0–100% B in 60 min at 1 mL/min. Collected fractions were analysed on a API150EX single quadrupole mass spectrometer (ABSciex), and those containing the expected molecular mass of 8383.5 Da, corresponding to the peptide with N-terminus pyroglutamate, were pooled and liophylised.

### Biological assays with cHH isoforms

Adult *A. leptodactylus*, with an average weight of 41±5,9 g, were obtained from a local dealer. The animals were kept in 120 L tanks with closed circuit filtered and thoroughly aerated tapwater, at room temperature. Animals were fed with fish pellets three times per week. Crayfish were anesthetized in ice for 15 min and then bilaterally eyestalk ablated 48 h before the start of the experiment, in order to avoid any possible interference due to the endogenous cHH. To assay the hyperglycemic activity, lyophilized RP-HPLC fractions of synthetic or native peptides were resuspended in 50 µL sterile phosphate buffered saline (PBS). Peptide concentration was determined by UV absorbance at 280 nm using calculated ε values of 14815 M^−1^ cm^−1^ for the peptide oxidized form. The extinction coefficient was computed using the ProtParam programme on the ExPASy server [48]. Each animal was treated with a peptide amount equal to 1.7 pmol/g live weight. Before injection, peptide samples were diluted with sterile PBS and 100 µL of this solution was injected. Animals were bled at 0, 1, 2, 4, 8 and 24 h after injection. Hemolymph glucose level was quantified using the glucose oxidase method (One Touch glucose test kit – Lifescan).

### Statistics

For glycemia elicited at the various times, the normality of data was checked with a Shapiro-Wilk test and homogeneity of variance for each time across groups and for each peptide across times were checked with a Bartlett test. Analyses were conducted using non-parametric statistics, i.e. Kruskal-Wallis rank sum test with post-hoc Wilcoxon rank sum test pairwise comparisons with Bonferroni correction, since the null hypothesis of the Shapiro Wilk and/or the Bartlett tests could not be rejected. The box and whiskers plots were drawn with the boxplot command. In the text, values are expressed as mean ± standard error. Statistical analyses were performed using R version 2.9.2 software [49].
